# Ameloblastic carcinoma of the mandible: a case report

**DOI:** 10.1186/s40902-023-00380-y

**Published:** 2023-04-27

**Authors:** Satoru Ogane, Arisa Fujii, Taiki Suzuki, Kazuhiko Hashimoto, Sadamitsu Hashimoto, Masayuki Takano, Akira Katakura, Takeshi Nomura

**Affiliations:** 1 Oral Cancer Center, Tokyo Dental College, 5-11-13 Sugano, Ichigawa Chiba, Japan; 2 Department of Plastic, Oral and Maxillofacial, Teiko University School of Medicine, 2-11-1 Kaga Itabashi-ku, Tokyo, Japan; 3 Department of Oral Pathobiological Science and Surgery, Tokyo Dental College, 2-9-18 Kandamisaki, Chiyoda-ku Tokyo, Japan; 4 Department of Oral Oncology, Oral and Maxillofacial Surgery, Tokyo Dental College, 5-11-13 Sugano, Ichikawa Chiba, Japan; 5 Division of Clinical Laboratory, Ichikawa General Hospital, Tokyo Dental College, 5-11-13 Sugano, Ichikawa Chiba, Japan; 6 Department of Biology, Tokyo Dental College, 2-9-7 Kandasurugadai Chiyoda-ku, Tokyo, Japan; 7 Department of Oral and Maxillofacial Surgery, Tokyo Dental College, 2-9-18 Kandamisaki-cho, Chiyoda-ku Tokyo, Japan

**Keywords:** Ameloblastic carcinoma, Ameloblastoma, Malignant odontogenic tumor

## Abstract

**Background:**

Ameloblastic carcinoma is a malignant form of ameloblastoma and a very rare odontogenic tumor. We report a case of ameloblastic carcinoma that occurred after removal of a right-sided mandibular dental implant.

**Case presentation:**

A 72-year-old female patient visited her family dentist with a complaint of pain around a lower right implant placed 37 years previously. Although the dental implant was removed with the diagnosis of peri-implantitis, the patient experienced dullness of sensation in the lower lip and was followed up by her dentist, but after no improvement. She was referred to a highly specialized institution where she was diagnosed with osteomyelitis and treated the patient with medication; however, there was no improvement. In addition, granulation was observed in the same area leading to a suspicion of malignancy, and the patient was referred to our oral cancer center. The diagnosis of squamous cell carcinoma was made after a biopsy at our hospital. Under general anesthesia, the patient underwent mandibulectomy, right-sided neck dissection, free flap reconstruction with an anterolateral thigh flap, immediate reconstruction with a metal plate, and tracheostomy. Histological analysis of the resected specimen on hematoxylin and eosin staining showed structures reminiscent of enamel pulp and squamous epithelium in the center of the tumor. The tumor cells were highly atypical, with nuclear staining, hypertrophy, irregular nuclear size, and irregular nuclear shape, all of which were suggestive of cancer. Immunohistochemical analysis showed that Ki-67 was expressed in more than 80% of the targeted area, and the final diagnosis was primary ameloblastic carcinoma.

**Conclusion:**

After reconstructive flap transplantation, occlusion was re-established using a maxillofacial prosthesis. The patient remained disease-free at the 1-year 3-month follow-up.

## Background

Ameloblastic carcinoma (AC) is a malignant form of ameloblastoma (AB) and has a low incidence, accounting for 0.3–3.5% of all odontogenic tumors [1]. The clinical features of AC are often atypical, with painless swelling seen in about 38% of cases [2, 3], and on imaging, it resembles clearly defined monocystic/polycystic types of AB, making it difficult to differentiate from AB via clinical and imaging findings [4, 5]. Therefore, histopathological diagnosis is important. We report a case of AC of the mandibular region, including a review of the literature.

## Case report

A 72-year-old female visited her family dentist in February 2020 for pain and bleeding in the right mandibular dental implant region. Under the diagnosis of peri-implantitis, periodontal therapy and antibiotics were administered, with no improvement. In November 2020, two dental implants in the right mandibular first and second molars were removed, and the following year, one dental implant in the right mandibular second premolar was removed. Dullness of the right lower lip appeared after removal of the implant and did not improve; therefore, in April 2021, she visited highly specialized medical institution and diagnosed osteomyelitis and prescribed medication, but there was no change in her symptoms. A tumor-like lesion with granulation surfaced from the right mandibular molar region, and the cytological diagnosis was class IV, squamous cell carcinoma (SCC). The patient was referred to our oral cancer center in June 2021. Her previous medical history included hypertension, hyperlipidemia, herniated disc, and subarachnoid hemorrhage (after clipping surgery in 2013). There was no history of smoking or alcohol consumption.

### Clinical findings

**Fig. 1:**
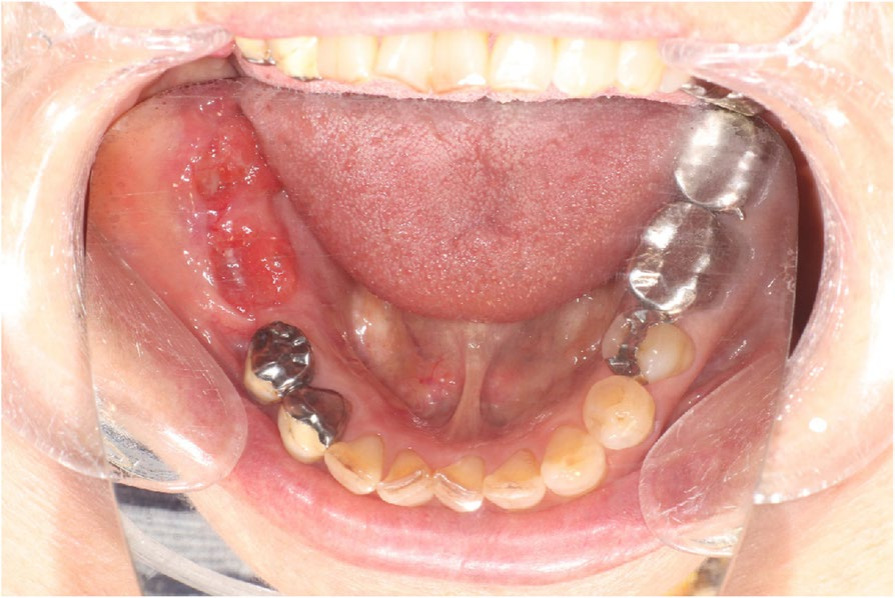
Intraoral picture showing erythematous growth of the mandible. The growth was approximately 30 mm in diameter on the right side of the mandible extending from #44 to #47. #44 was not mobile

Intraoral examination revealed a coarse granulomatous mass with a 30-mm-diameter ulcer on the gingiva, extending from the right mandibular first premolar to the right mandibular second molar (Fig. 1). However, #44 was not mobile. Extraoral findings were a symmetrical facial appearance and no swollen lymph nodes.

### Imaging findings

**Fig. 2:**
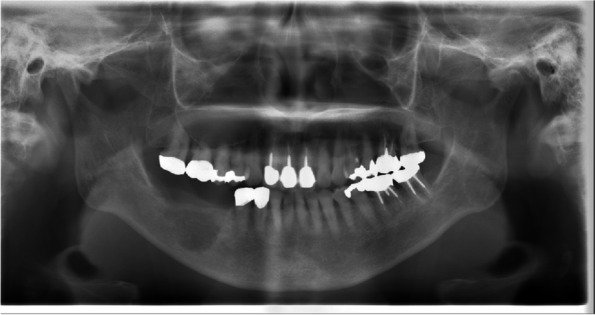
Preoperative panoramic radiography. An ill-defined radiolucent lesion extending from the mesial aspect of #45 to the equivalent of #47. The mandibular canal was indistinct

**Fig. 3:**
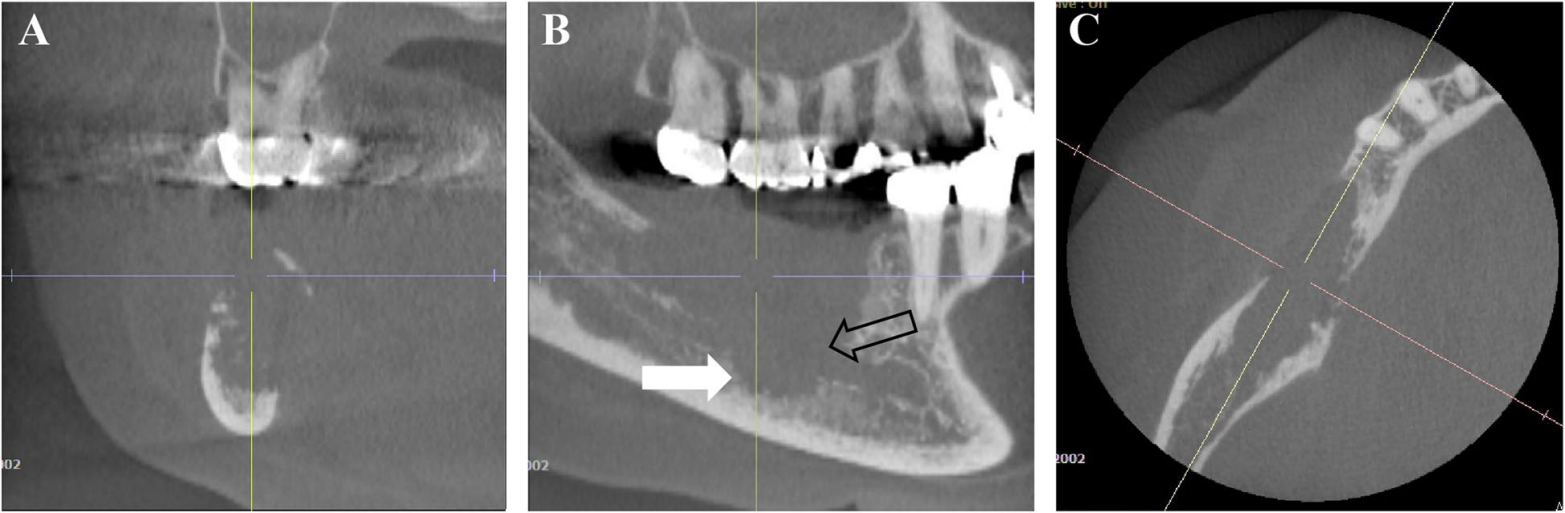
Preoperative CT images and cone beam computed tomography (CBCT). Coronal (**a**), axial (**b**), and sagittal (**c**) cone-beam computed tomographic images show multilocular cortical expansion (arrows) and the indistinct mandibular canal (open arrows)

**Fig. 4:**
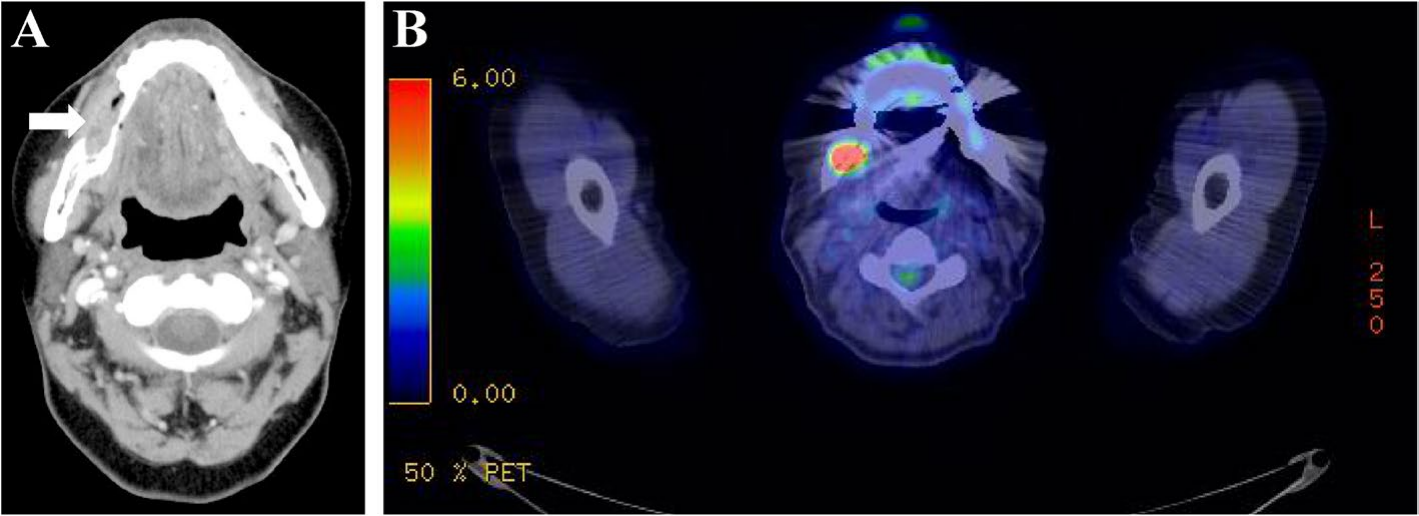
Preoperative CT image and PET-CT. Enhanced axial computed tomographic images (**a**) showing a large tumorous mass pushing out towards adjacent soft tissue bucco-lingually. Inside the tumor, necrotic foci (arrows) can be observed. PET-CT (**b**) showing a mass protruding on the bucco-lingual side in the #45 and #46 region with FDG accumulation and SUVmax of 22.73

**Fig. 5:**
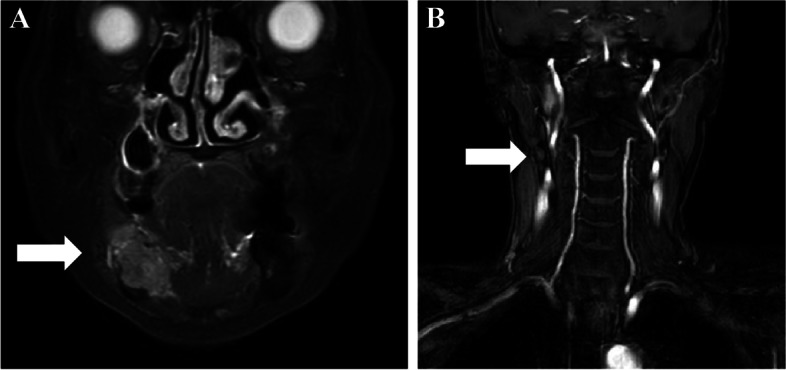
Preoperative MRI images. Enhanced coronal magnetic resonance images showing **a** a lesion of approximately 35 × 18 × 26 mm in size and **b** a level III lymph node swelling on the right side

Panoramic radiography showed irregular marginal multifidus bone resorption and multilocular radiolucency; bone resorption extended from the right mandibular first premolar to the area corresponding to the right mandibular second molar, there was no tilting of the tooth axial inclination, and the mandibular canal was indistinct (Fig. 2). Cone-beam computed tomography (CT) showed a radiolucent depicting frank bone destruction with residual bone remnants and multilocular, expansile cortical resorption (Fig. 3A–C). Contrast enhanced CT revealed the lesion as a mass with contrast effect measuring of approximately moderately 23 × 18 × 22 mm (Fig. 4A). Bone erosion was observed in region of the mandibular canal and cortical bone on the buccolingual side, with partial infiltration into the floor of the oral cavity. Positron-emission tomography (PET)-CT also showed a buccal protruding mass with fluorodeoxyglucose (FDG) accumulation of standard uptake value (SUV) max 22.73, with bone penetrating images consistent with the lesion (Fig. 4B). MRI showed the lesion to be a mass of approximately 35 × 18 × 26 mm. The lesion showed a low signal on T1-weighted images, an intermediate signal on T2-weighted images, a strong high signal on diffusion-weighted images, a low signal on apparent diffusion coefficient (ADC) map, and a heterogeneous and mild contrast effect on dynamic contrast-enhanced MR images (Fig. 5A). In addition, there were findings suggestive of level III lymph node metastasis on the right side (Fig. 5B).

There were no other findings of cervical lymph node metastasis or distant metastasis.

#### Laboratory findings and preoperative biopsy findings

**Fig. 6:**
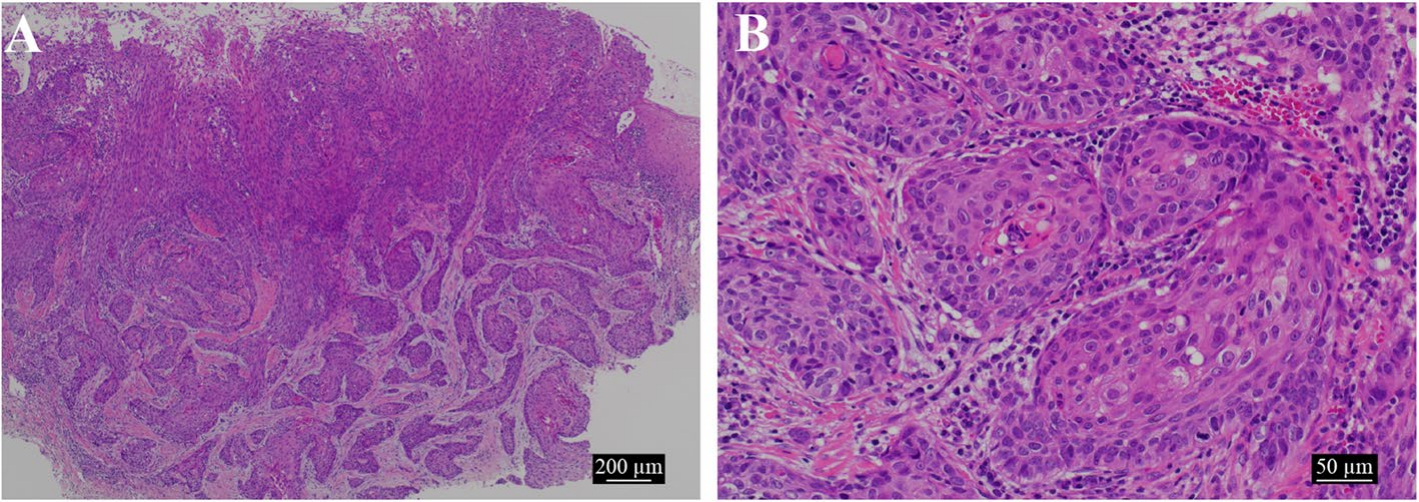
Histological examination of biopsy. **a** An image showing atypical epithelium proliferating and forming small and large foci × 40. The tumor is partially exposed and ulcerated, and areas of continuity with the coated epithelium can be observed. **b** The lesions show a keratinizing tendency and some appear fenestrated (× 200). Tumor cells showing strong atypia, including nuclear staining and enlargement, discrepancy in nuclear size, and irregularity in nuclear shape

Blood tests, electrocardiography, pulmonary function tests, and chest radiography showed no abnormal findings. Biopsy was performed in June 2021, and a diagnosis of squamous cell carcinoma (SCC) was made. At 40 × , atypical epithelium is proliferating, forming large and small foci. The superficial layer is ulcerated with exposed tumor, and at the right edge, the tumor and coated epithelium are continuous. At 200 × , the foci show a keratinizing tendency, and some of them appear to be fenestrated. Tumor cells show strong atypia, including nuclear staining and enlargement, nuclear size disparity, and irregular nuclear shape. At this point, the diagnosis was squamous cell carcinoma (Fig. 6A, B).

#### Clinical course

**Fig. 7:**
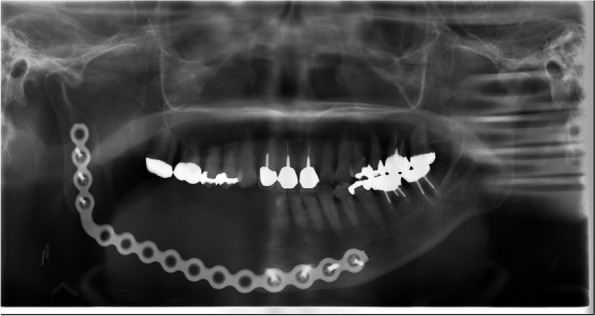
Panoramic radiographs on postoperative day 8. The image findings confirmed that the plate was intact

Under general anesthesia, the patient underwent mandibulectomy, right-sided neck dissection, free flap reconstruction with an anterolateral thigh flap, immediate reconstruction with a metal plate, and tracheostomy. The reason we had not selected a fibular graft was due to the possibility of osteonecrosis of the jaw due to post-operative therapy because of the preoperative diagnosis of SCC. A preoperative diagnosis of malignancy was made, and a safety margin of 10 mm was established around the tumor according to the resection method for malignant tumors; the inferior alveolar nerve bundle was resected. Regarding the extent of prophylactic neck dissection, we decided to perform SND (I・II・III). On the 19^th^ day, she was discharged. It has been 1 year and 3 months since the surgery, with no recurrence or distant metastasis (Fig. 7).

#### Postoperative pathological findings

**Fig. 8:**
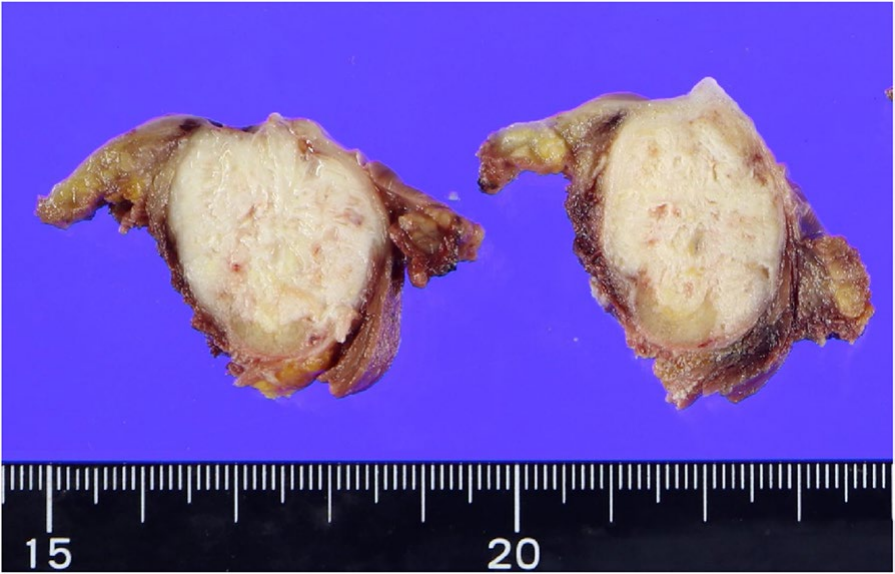
A relatively well-defined white, white, fulfilling tumor filling the jawbone

**Fig. 9:**
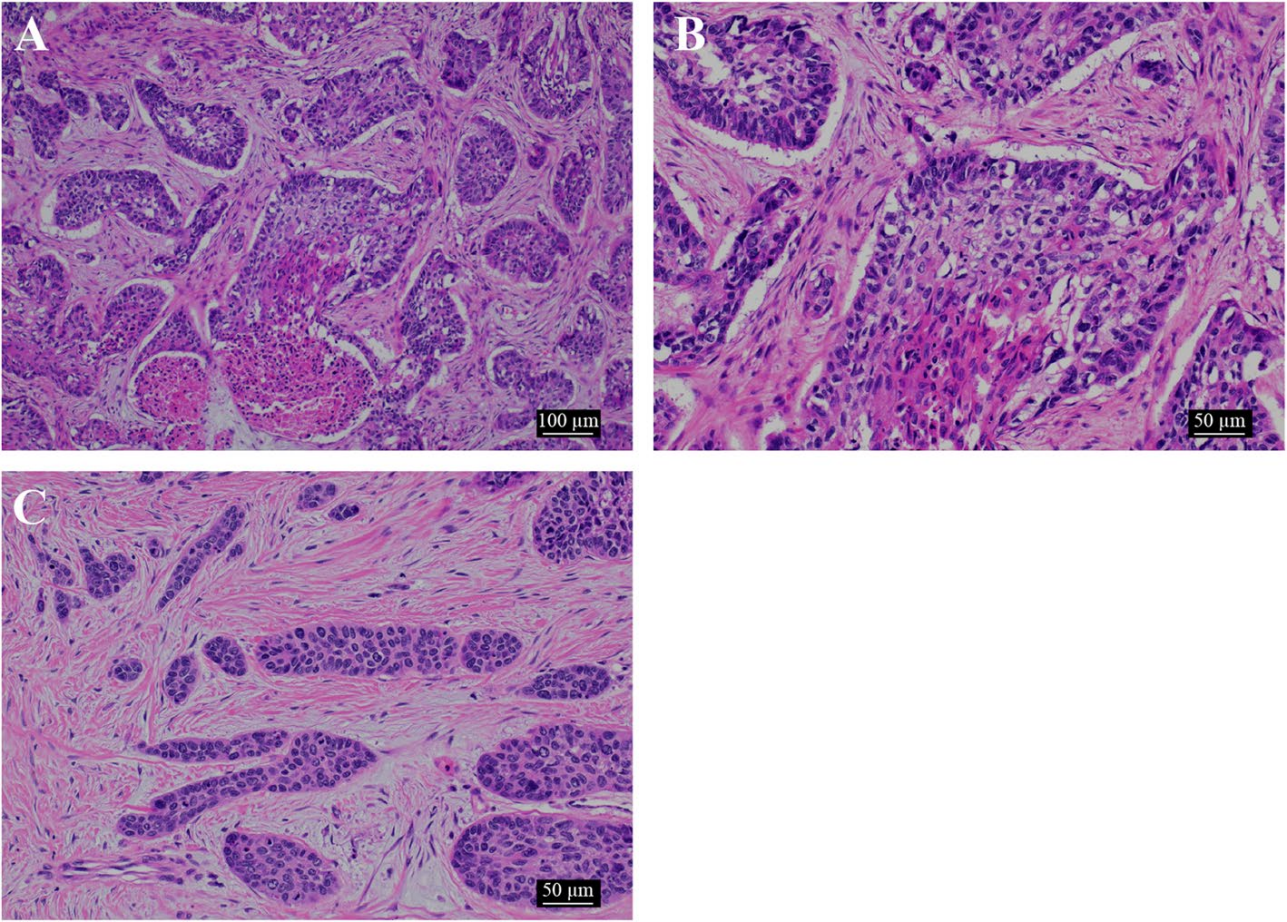
Histological examination. **a** Part of the atypical epithelium is proliferating, forming small and large foci with necrosis (× 100). **b** The margins of the tumor foci show a fenestrated arrangement, and enamel pulp-like structures and squamous metaplasia are seen in the center of the foci. It is accompanied by a high degree of atypia (× 200). **c** Some parts of the lesion grow while forming epithelial cords and epithelial islands, which resemble dental crests (× 200). The position of the ameloblastic carcinoma is also visible

**Fig. 10:**
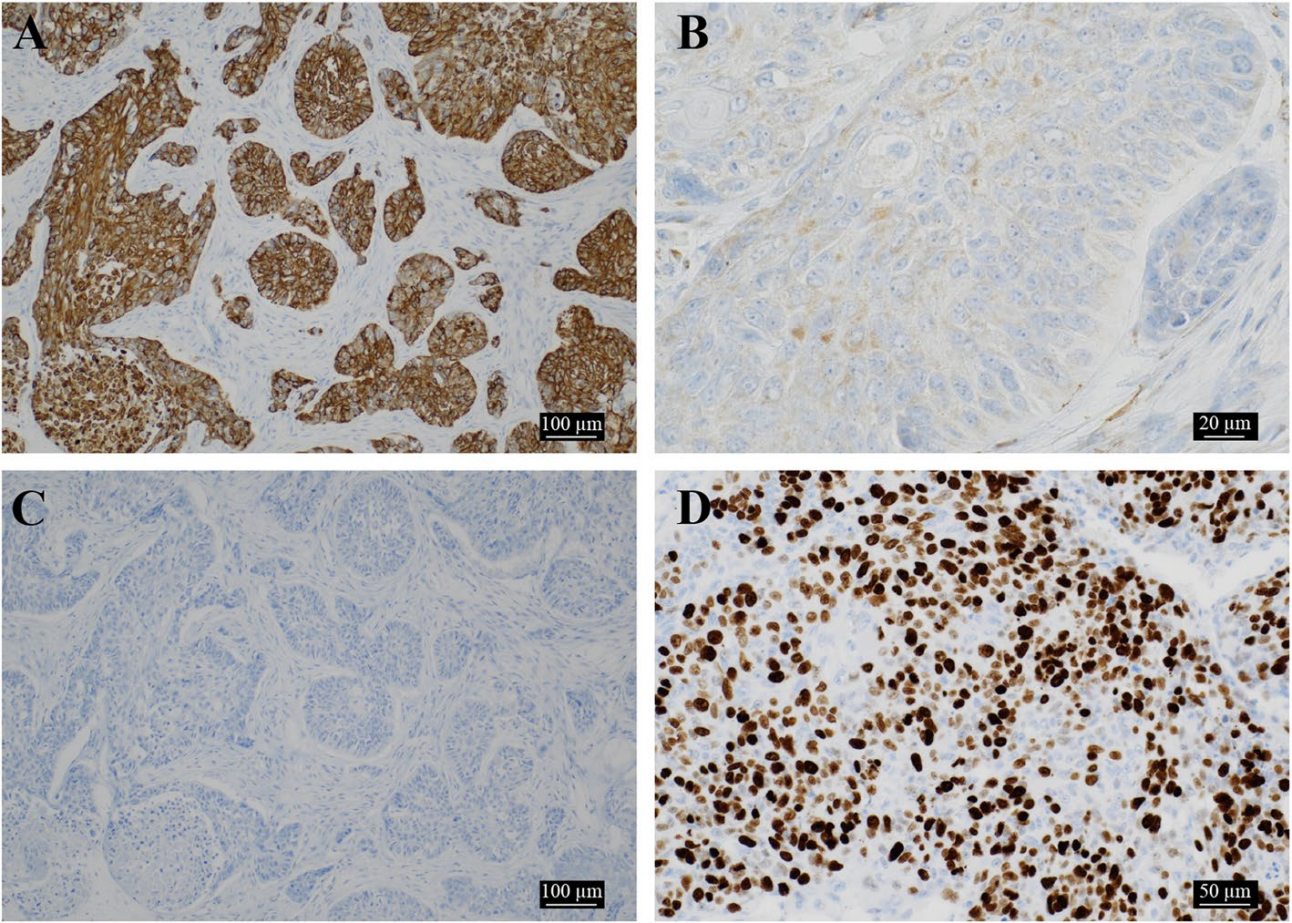
Immunohistochemical evaluation. **a** Cytokeratin 19 is positive (× 100). **b** α-SMA is partially weakly positive (× 400). **c** p53 is negative (× 100). **d** Many tumor cells are positive for Ki-67 (× 200). The position of the ameloblastic carcinoma is visible

The tumor was moderate to partially atypical and proliferated in the connective tissue, forming alveolars. A palisading of tumor cells was observed on the basal side of the foci, with stellate cell like cell proliferation, keratinization, squamous metaplasia, blanching, and central necrosis. (Figs. 8 and 9A–C). We observed destruction of the cortical bone on the buccolingual side, extraosseous infiltration, and tumor infiltration into the musculature of the lingual side, but no marginal exposure. Furthermore, no invasion was seen along the surrounding salivary glands and inferior alveolar nerve. Immunohistochemical staining was positive for the odontogenic epithelial marker CK19, weakly positive for α-SMA in some areas, negative for p53, and positive for Ki-67 in more than 80% of the hot spot areas, with the final diagnosis being primary AC (Fig. 10A–D).

## Discussion

According to the 2005 WHO classification, the treatment strategy for primary AC is the same as that for the secondary (dedifferentiated) endosteal/periosteal type, and the advanced secondary type is difficult to distinguish histopathologically from the primary type. Therefore, the 2017 classification was revised to a single disease classification [6–8]. The incidence of AC is reported as 11 cases (0.21%), out of 5,231 odontogenic tumors, and in other countries, it is reported to account for approximately 1.6–2.2% of all odontogenic tumors [9–11]. The average age of AC incidence is 45.9–49.4 years, males are approximately twice at risk compared to females, and involvement of the mandible is approximately twice as common as that of the maxilla [12, 13]. Clinically, AC is more destructive and invasive than ameloblastoma (AB). It is also characterized by a variety of symptoms including swelling with rapid growth, cortical bone perforation, pain, ulcer or fistula formation, facial asymmetry, dysphagia, and dysesthesia [2–5]. However, approximately 38% of ACs may be characterized only by painless swelling and lack typical clinical symptoms [2, 3]. Furthermore, the diagnosis rate of preoperative AC is reported, invasion as low as 39%, and preoperative diagnosis is considered difficult [14]. In the past 10 years, 13 (61.9%) of 21 cases of AC in the area of the bone of the jaw were diagnosed as benign tumors (Table 1) [15–31]. On the other hand, 7 cases (33.3%) led to a diagnosis of a malignant tumor. In this case, the preoperative diagnosis was SCC. The CT images of ACs are often characterized by malignant tumor images with indistinct borders, bone destruction, invasion into the surrounding soft tissues [2, 32, 33]. However, similar to ABs, ACs are often associated with well-defined, single/multifocal radiographic findings [4, 5]. In this case, CT images showed bone disruption and soft tissue invasion in the surrounding area. MR images showed a strong high signal on diffusion-weighted images, and the tumor parenchyma could be identified. Histopathologic features of AC are pleomorphism, fission, focal necrosis, perineural invasion, and hyperchromasia of the nuclei, in addition to the histopathologic findings of AB. Hall et al. reported the pathological findings of general AC to be the absence of a central stellate reticular region and the presence of epithelial cell aggregates, cytosolic plexiform or insular, hyperchromatin, dense cellular arrangement, atypical nucleoli, focal necrosis, and neural and vascular invasion [34], which can make it difficult to distinguish from AB. Therefore, immunochemical staining is used to differentiate ACs from ABs. In particular, Ki-67, p53, and α-SMA are known to be useful markers [33, 34]. Ki-67 and p53, markers of tumor cell proliferative activity, are highly expressed in AC. Kase et al. proposed diagnostic criteria for AC as a Ki-67 positivity rate of 10% or higher [35]. In addition, ACs with clear cells have anaplastic features and are more invasive than ACs without clear cells and have higher recurrence and mortality rates [36, 37]. In this case, there were no clear cells, and the tumor cells formed foci with stellate cell like cell proliferation, keratinization, squamous metaplasia, vacuolation, and blister necrosis; lymphatic invasion was also observed. Ki-67 showed more than 80% expression in the affected area, and AC was finally diagnosed. The occurrence of AC has been considered attributable to the contact of malignant epithelial tumors with calcified, dentin-like, and bone-like hard tissue formations, and the tumor epithelium of AC is thought to have an inductive effect on the tumor mesenchyme [38]. Other reports are suggesting that the remaining tissue of the tooth embryo may have originated from the entrapped salivary gland epithelium, but this remains to be clarified [31, 38]. Surgical resection is the first choice of treatment for ACs, and the efficacy of radiation chemotherapy has not been established [31, 35]. There is no association between prophylactic neck dissection and improved survival, and postoperative radiation therapy has been shown to be beneficial in cases of invasion into the surrounding soft tissue or positive margins [39]. In this case, the preoperative diagnosis was SCC (rT4aN0M0); therefore, prophylactic neck dissection was performed with a safety margin of 10 mm according to the procedure for malignant tumors. Intraoperative rapid diagnosis confirmed the negative margins; the final preparation also had negative margins, and there were no metastatic findings in the excised lymph nodes.

**Table 1:** Reports of AC in the jawbone region in the last 10 years

First symptoms			Imaging findings			Clinical diagnosis	Cytological diagnosis	Treatment	Metastases/ recurrence(mon)	Outcome
Pain	Swelling	Others	Form	Bone destruction	Invasion					
–	+	Dysphagia, dysarthria	Multifocal	+	–	AB	AC	Subtotal resection	Unknown	Unknown
+	+	Ulcer	Single	+	–	Malignant tumor	AC	Hemi-mandibulectomy + RT	Unknown	Unknown
–	+	Tooth mobile	Single	+	–	Dentigerous cyst	–	Excision	–	Survive (24 M)
+	+	–	Multifocal	+	–	AB	AC	Hemi-mandibulectomy	Unknown	Unknown
–	+	Paresthesia, tooth mobile	Unknown	–	+	AB	AC	Segmental resection	–	Survive (18 M)
+	+	induration	Multifocal	+	–	AB	AB,SCC	Hemi-mandibulectomy	–	Survive (19 M)
–	+	–	Multifocal	Unknown	Unknown	AB	AC	Segmental resection	Unknown	Unknown
–	+	–	Single	+	–	AB	Malignant odontogenic tumor	Subtotal resection	–	Survive (22 M)
+	–	Infection	ingle	+	–	Dentigerous cyst	AC	Segmental resection + RT	Unknown	Unknown
–	+	–	Multifocal	+	–	AB	AC	Hemi-mandibulectomy	–	Survive (16 M)
+	+	Nasal obstruction, dysarthria	Unknown	+	+	Malignant tumor	–	Segmental resection	–	Survive (12 M)
–	+	Abnormal sockets of extracted tooth	Multifocal	+	+	Malignant tumor	Spindle cell carcinoma	Segmental resection + RT	Unknown	Survive (13 M)
–	+	Ulcer	Single	Unknown	Unknown	Fibroma	Unknown	Unknown	Unknown	Unknown
–	+	Nasal obstruction	Unknown	+	+	Malignant tumor	AC	RT	–	Survive (120 M)
–	+	–	Single	Unknown	–	AB	Basaloid squamous cell carcinoma	Hemi-mandibulectomy	–	Survive (6 M)
–	–	Dysphagia	Multifocal	+	+	Malignant tumor	–	Segmental resection	–	Survive (4 M)
+	+	Nasal obstruction	Unknown	–	–	Benign lesion	AC	Resection + CRT	Unknown	Unknown
–	+	Paresthesia	Single	+	–	Benign tumor	–	Subtotal resection	Unknown	Unknown
–	+	–	Multifocal	+	+	Malignant tumor	–	Segmental resection	–	Survive (36 M)
–	–	Paresthesia	Multifocal	+	+	Malignant tumor	SCC	Segmental resection	–	Survive (15 M)

The 5-year survival rate of AC is 69.1–83.2%, which is relatively high [39–41], but it decreases to 0–21.4% in metastatic cases [39, 40], and the recurrence rate is said to be 20.9–38.4% [12, 32, 41]. As for distant metastases, the lungs are the predominant site, with an incidence of 15.4–22.0% [32, 42, 43]. A characteristic feature of AC is the long time until recurrence or distant metastasis: the time to recurrence is 47.5 months, and the time to distant metastasis is 84.7 months, although cases occurring after 156 months have also been reported [40]. Therefore, long-term follow-up of ACs is important [39, 40], and Jaitley et al. recommend CT evaluation every 6 months [43].

Recent developments in molecular biological techniques have improved tumor therapy. Osteogenic tumors are associated with a high incidence of BRAF-V600E mutations, which have been suggested to be associated with AB invasion [42, 44–47]. Two targeted agents, dabrafenib, which blocks the action of BRAF mutations, and trametinib, a MEK inhibitor, have been reported to be effective in patients with ABs and lung metastases [48]. In the future, targeted drug therapy for ACs with BRAF-V600E mutations is suggested. In this case, there was no suspicion of recurrence or metastasis on CT at 6 months postoperatively. However, it is necessary to perform long-term local and systemic follow up in such cases.

## Data Availability

Not applicable.

## Abbreviations

AB: Ameloblastoma
AC: Ameloblastic carcinoma
ADC: Apparent diffusion coefficient
CT: Computed tomography
FDG: Fluorodeoxyglucose
HE: Hematoxylin eosin
MR: Magnetic resonance
PET: Positron emission tomography
SCC: Squamous cell carcinoma
SUC = VG: Standard uptake value
